# Quantification of Differential Metabolites in Dried Blood Spots Using Second-Tier Testing for SCADD/IBDD Disorders Based on Large-Scale Newborn Screening in a Chinese Population

**DOI:** 10.3389/fped.2021.757424

**Published:** 2021-11-19

**Authors:** Wei Zhou, Heng Cai, Huizhong Li, Zhe Ji, Maosheng Gu

**Affiliations:** ^1^Newborn Screening Center, The Affiliated Xuzhou Maternity and Child Health Care Hospital of Xuzhou Medical University, Xuzhou, China; ^2^Research Center for Biochemistry and Molecular Biology and Jiangsu Key Laboratory of Brain Disease Bioinformation, Xuzhou Medical University, Xuzhou, China; ^3^Pharmacology College, Xuzhou Medical University, Xuzhou, China; ^4^The First Clinical College, Xuzhou Medical University, Xuzhou, China

**Keywords:** SCADD, IBDD, second-tier screening, differential detection, UPLC-MS/MS

## Abstract

**Background:** Although newborn screening (NBS) for metabolic defects using the marker butyl carnitine (C4) combined with the C4-to-acetylcarnitine ratio is adequate, the incorporation of novel parameters may improve differential testing for these disorders without compromising sensitivity.

**Methods:** Analytical and clinical performance was evaluated by MS/MS using 237 initially positive neonatal samples between March 2019 and March 2020 at the Newborn Screening Center of Xuzhou Maternity and Child Health Care Hospital. Additionally, second-tier testing by ultraperformance liquid chromatography-tandem mass spectrometry (UPLC-MS/MS) combined with the quantification of ethylmalonate (EMA) or isobutyryl-glycine (IBG) in dried blood spots (DBSs) was performed to reduce the false-positive rate.

**Results:** We reviewed initial MS/MS data for DBSs from 469,730 neonates, and a second-tier test was performed using 237 samples that exceeded the C4 concentration cutoff value. Eleven variants of the *ACADS* gene were identified, with c.1031A>G (p.E344G) being the most common. Fifteen *ACAD8* mutations were identified in seven patients, and Swiss modeling and amino acid conservation analyses were conducted for the novel variants. Based on a retrospective analysis of EMA and IBG, the application of second-tier tests before the release of neonatal screening results reduced referrals by over 91.89% and improved the positive predictive value (PPV) for short-chain acyl-CoA dehydrogenase deficiency/isobutyryl-CoA dehydrogenase deficiency (SCADD/IBDD) screening.

**Conclusion:** A screening algorithm including EMA/IBG improves target differential testing for NBS and may eliminate unnecessary referrals while maintaining 100% sensitivity. Second-tier screening using UPLC-MS/MS as a rapid and convenient supplemental DNA sequencing method may be beneficial for differential detection.

## Introduction

Acylcarnitine profiling of dried blood spots (DBSs) by tandem mass spectrometry (MS/MS) is a valuable measurement tool for high-throughput newborn screening (NBS), which is important for diagnosing deficiencies, including those in fatty acid β-oxidation (FAO) and branched-chain amino acid metabolism (1). Unfortunately, this method is utilized without the differentiation of acylcarnitine into isomers, isobaric compounds, and contaminants based on non-derivatization MS/MS. Despite the development of several high-performance liquid chromatography-MS/MS methods for separating isomers in serum and urine, such approaches remain time-consuming, involve different derivatization steps, or focus on specific acylcarnitines. However, a new ultraperformance liquid chromatography (UPLC–MS/MS) method that assesses special analytes as markers for short-chain acyl-CoA dehydrogenase deficiency (SCADD), isobutyryl-CoA dehydrogenase deficiency (IBDD), and other disorders has been developed (1, 2).

Previous studies have shown that analyte testing for butyl carnitine (C4) in DBSs is able to confirm SCADD (MIM 606885) and IBDD (3) (MIM 611283), and this assay is also used to test for ethylmalonic encephalopathy (EE) (4, 5) (MIM 201470), glutaric acidemia type II (GA2) (6, 7) (MIM 213680), and formiminoglutamic aciduria (FIGLU, MIM 229100). Nevertheless, increasing concentrations of methylsuccinic acid (MSA) concentration serves as a special biochemical marker for EE (8), and elevations in OX-2-acetoacetic acid, isovalanyl glycine-2,2-hydroxyisobutyric acid-2, and adipic acid to extremely high levels are observed in GA2 (9). In some cases of FIGLU, formiminoglutamate is observed in biological fluids after histidine loading (10). Therefore, a novel second-tier screening method involving the use of UPLC-MS/MS can detect the secondary targets ethylmalonic acid (EMA) and isobutyryl-glycine (IBG), which are part of the differential diagnosis or profile review of screening for primary targets (11, 12). According to Adhikari et al., second-tier biochemical testing excludes nearly 50% of initial positive MS/MS tests (13).

Short-chain acyl-CoA dehydrogenase deficiency is an autosomal recessive inborn-error metabolic disease of mitochondrial FAO (3, 14–16). The pathogenic gene is traditionally considered to be *ACADS*, and the biochemical signatures of defects in short-chain acyl-CoA dehydrogenase (SCAD) result in the accumulation of plasma C4 and urine EMA (17, 18). Extremely rare autosomal recessive metabolic disorder IBDD has a heterogeneous phenotype that generally exhibits a mild clinical course (19–21). The first case of an inborn defect in the IBD gene was detected in 1998, and an increased level of urinary IBG has been reported (20). Many individuals with confirmed IBDD either show mild manifestations or are asymptomatic, which is comparable to classic organic academia (22). The natural history of SCADD and IBDD was elucidated in recent years via NBS and clinical follow-up of individuals with abnormal screening results (13). Although SCADD and IBDD typically involve benign biochemical phenotypes, additional factors, including environmental effects, associated genes, and the accumulation of toxic metabolites, may participate in the progression of their disorders (23). Therefore, to avoid misdiagnosis, novel second-tier testing programs have been reluctant to halt evaluations of neonates with elevated C4 concentrations.

The present study assessed the correlation of the initial DBS C4 value and the second-tier DBS EMA and IBG value in a large cohort of individuals identified as potential cases of SCADD/IBDD through NBS. We investigated whether the combined consideration of multiple test results improves the specificity of NBS for the differentiation of SCADD/IBDD from other disorders in an effort to reduce the burden of follow-up testing and unnecessary anxiety for families.

Although previous publications have described the familial impacts of NBS results, but these studies did not focus specifically on SCADD and IBDD within the context of benign clinical findings. Thus, to contribute to current data on these conditions, we evaluated the clinical biochemical outcomes of infants who were identified by the NBS program as having SCADD or IBDD from 2015 to 2020 and compared them to the health status of an age-matched control group. We also performed phone interviews to collect statements from parents on their experiences with the NBS and the clinical diagnosis of their children with SCADD/IBDD (24).

## Materials and Methods

Among 469,730 neonates (249,032 males and 220,698 females) recruited at the Newborn Screening Center of Xuzhou Maternity and Child Health Care Hospital between November 2015 and March 2020 and analyzed by MS/MS throughout the study period, increased C4 levels and relative ratios that exceeded the cutoff values were found in 1,029 neonates (0.219%, 1,029/469,730). These results were considered to be initial positive screening results for SCADD, IBDD, and other metabolic disorders. From March 2019 to March 2020, 237 neonatal samples positive for C4-associated metabolic defects based on elevated C4 and related ratios were collected by using a second-tier test by UPLC-MS/MS test. Moreover, urinary GC/MS was performed as a common detection method that would be used with recall of positive individuals. Mutation analysis, which involved sequencing was performed for diagnosis, and “site validation” of variants relevant to the screened disorders was performed. A genetic family tree was constructed using data from parental peripheral blood leukocytes obtained via Sanger sequencing. DNA sequencing was performed using an ABI-3100 automated sequencer (Applied Biosystems, Foster City, CA, USA), and the DNA sequencing pipeline developed for screening purposes, which included the *ACADS* and *ADAD8* genes as alternative targets, was employed for the clinical diagnosis of individuals identified as positive by MS/MS screening. The DNA panel used in our center contained 76 genes divided into two groups, and these 76 genes were curated based on evidence for their association with metabolic conditions in the Xuzhou area (Supplementary Table 1). Evaluations by the quality assurance program of the China National Center for Clinical Laboratories consistently yielded 100% satisfactory results between 2015 and 2020. The Research Ethics Board of the Xuzhou Maternity and Child Health Care Hospital approved this study.

For this study, we developed an additional biochemical informatic analysis to assess whether such analyses aid in case resolution without the need for follow-up metabolic testing. Variant bioinformatics analysis was implemented as previously described, and the identified variants were searched in databases (23). The pathogenicity of missense variants was evaluated using the PolyPhen-2 tool, and evolutionary conservation was assessed via ClustalX (http://www.clustal.org/clustal2). Swiss Model Workspace was used for homology modeling to analyze changes in three-dimensional (3D) structures.

### Second-Tier Biochemical Assay

Samples from three DBSs (*d* = 3.2 mm) (the DBSs were stored at 4°C) were punched into a 96-well plate, and 100 μl of a liquid containing internal standards was added to yield a methanol, water and formic acid at a ratio of 50:49:0.42. Briefly, the mixture was rotated at 600 rpm and 35°C for 30 min and rested for 5 min to stop the reaction and methanol was removed under heated nitrogen flow at 55°C. The resulting residue was reconstituted in 50 μl of water. The samples were separated using a Kinetex 2.6 μm XB-C18 100A column (Phenomenex, USA) with a Waters ACQUITY UPLC system (Waters, USA) coupled to a Xevo XE tandem mass spectrometer (Waters, USA). Supplementary Table 2 shows the parameter settings for the second-tier analyte testing. UPLC-MS/MS was operated in the multiple reaction monitoring (MRM) negative mode to follow precursor-to-product species transitions for EMA (130.98–86.96 m/z) and IBG (143.95–73.8 m/z) as well as the corresponding internal standards by negative ion electrospray ionization (ESI)-MS/MS and a mass-to-charge ratio (Supplementary Table 3) (25). The total run time was 8.5 min per sample. The peak and results for the EMA/IBG internal standard are shown in **Figure 4F**.

### Statistical Analysis

According to the guidelines of the Clinical and Laboratory Standards Institute (CLSI) guidelines (EP28-A3c: Defining, Establishing, and Verifying Reference Intervals in the Clinical Laboratory, Approved Guideline, 3rd edition), the D/R ratio was ≤1/3, and the two values were not outliers. Cases that lacked quantitative data (fewer than 5,000 cases) were assessed for a normal distribution using the Kolmogorov-Smirnov (K-S) test. Significant data (*p*-value <0.05) did not adhere to a normal distribution, as demonstrated by K-S testing, and the percentile of the non-parametric test percentile was used to establish the cutoff. Throughout the entire study period, cases with elevated C4 concentrations at the initial NBS were recruited for second-tier detection by UPLC-MS/MS. A total of 2,308 samples from healthy individuals as the negative group were chosen to calculate the cutoff value using the percentile distribution of metabolites.

## Results

### C4 Acylcarnitine Determination by MS/MS for Initial NBS

**Figure 1:**
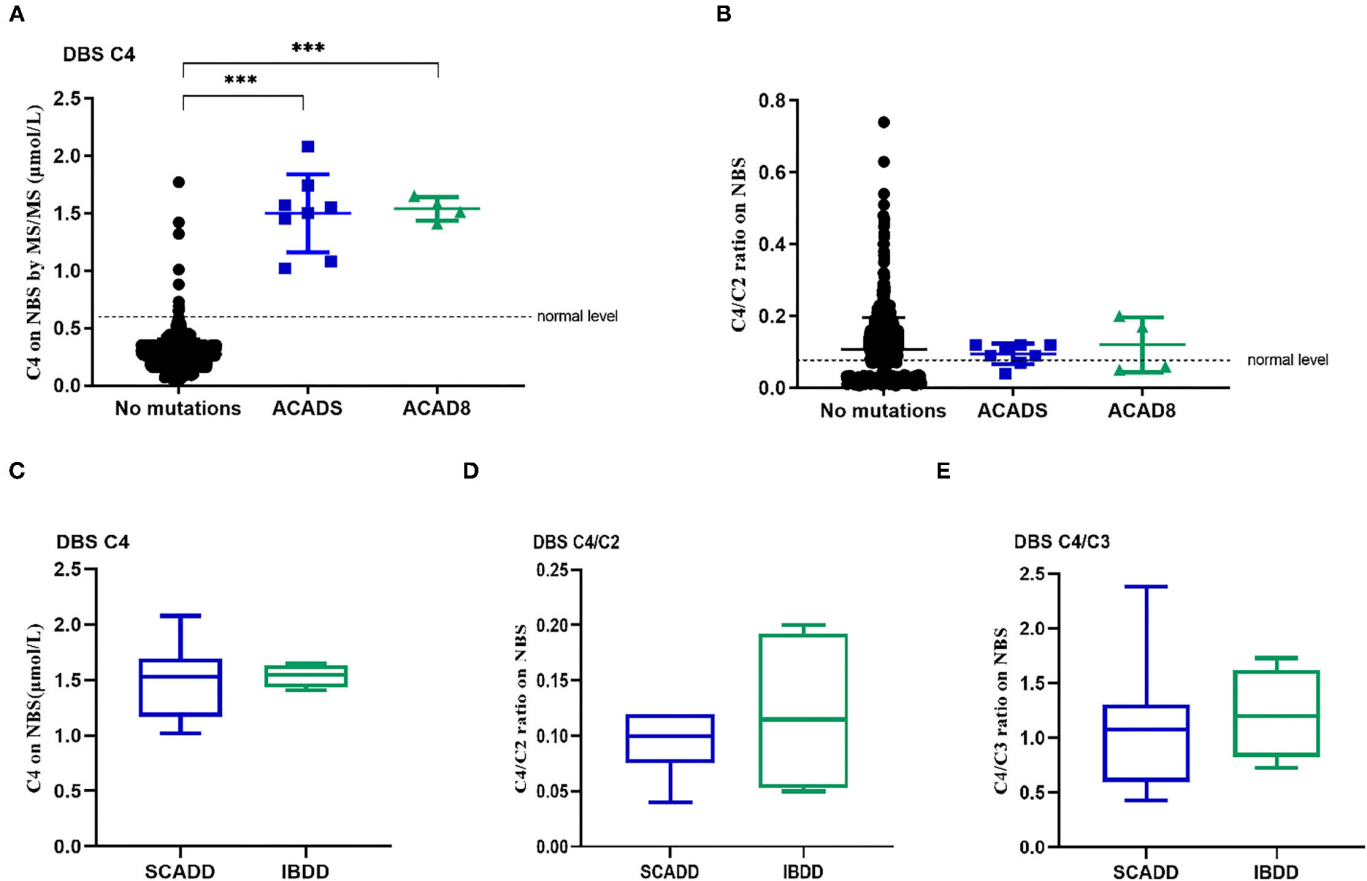
Metabolite levels in individuals with molecularly confirmed *ACADS* and *ACAD8* variants. **(A)** Levels of C4 identified by newborn screening. **(B)** C4/C2 ratios obtained from newborn screening. **(C–E)** Box plots of biochemical tests using MS/MS for SCADD compared to IBDD. **(C)** C4-acylcarnitine concentrations identified by newborn-screening blood spots (DBSs C4). **(D)** C4/C2 ratios in DBSs. **(E)** C4/C3 ratios in DBSs. (^*^p < 0.05; ^**^p < 0.01; ^***^p < 0.001).

The major biochemical characteristics identified throughout the study period are summarized in Table 1 and Figure 1, and these including the corresponding C4, C4/C2, and C4/C3 levels obtained by MS/MS. Figures 1A,B show the scatter plots for the C4 vs. C4/C2 ratios and differences among SCADD, IBDD, and no-gene mutation cases. In the initial NBS approach, the cutoff value for C4 was 0.04–0.42 μmol/L. The mean plasma concentrations of C4 in individuals with SCADD and individuals with IBDD were 1.42 and 1.39 μmol/L, respectively. Further statistical analysis revealed a significant difference between individuals with SCADD or IBDD and the no-mutation group (*p* < 0.001). The C4/C2 and C4/C3 ratios were also above the cutoff values in individuals with confirmed SCADD or IBDD, and compared with the no-gene-mutation group, the mean C4/C2 or C4/C3 ratios of the confirmed cases obtained from the initial NBS by MS/MS were nearly double the cutoffs. However, no significant difference was found between the SCADD and IBDD groups.

**Table 1:** Levels of abnormal parameters for the two associated different disorders with C4 by MS/MS and urinary GC/MS.

**Total cases** **(***n*** = 12)**	**Short-chain acyl-CoA dehydrogenase deficiency (SCADD)**		**Isobutyryl-CoA dehydrogenase deficiency (IBDD)**	
	**Initial screening**	**Recalling screening**	**Initial screening**	**Recalling screening**
*N* (%)	66.7		33.3	
**Plasma abnormal parameters by MS/MS**				
C4 Concentration mean (range), μmol/L (0.06–0.45)	1.50 (1.02–2.08)	1.43 (0.61–2.06)	1.54 (0.72–1.65)	1.56 (1.21–2.48)
C4/C2 mean ratio (0–0.04)	0.095 (0.04–0.12)	0.13 (0.07–0.22)	0.12 (0.05–0.20)	0.14 (0.10–0.24)
C4/C3 mean ratio (0.04–0.4)	1.12 (0.43–2.38)	1.51 (0.84–2.48)	1.22 (0.73–1.73)	1.49 (0.91–2.95)
**Urinary abnormal parameters by MS/MS**				
EMA (0–6.2 μmol/L)	13.57 (2.96–26.55)		0.57 (0–1.20)	
IBG (0–0.4 μmol/L)	0.00		0.82 (0.03–1.79)	

### Biochemical and Molecular Genetic Descriptions of Individuals With SCADD or IBDD

We developed a local NBS flow for the diagnosis of inherited metabolic diseases in Xuzhou, China, and recall of positive individuals is recommended for further detection by urinary GC/MS and DNA sequencing. Among 17 cases (4 females and 13 males) that were analyzed in Xuzhou, China, many involved pathogenic/likely pathogenic (P/LP) variants in associated genes (Table 2); a small number were variants of uncertain significance (VUSs). In total, positive urinary GC/MS results were found for eight infants with SCADD and fur with IBDD identified with two P/LP variant or one P/LP and one VUS variant in a pathogenic gene. Other cases were suspicious for SCADD/IBBD and were followed up in our center for a long time after birth.

**Table 2:** Molecular characteristics for the SCADD and IBDD individuals identified with two pathogenic variants.

**No**.	**Gender**	**Allele 1**		**Allele 2**		**Other variant**		**Type of mutation**	**Pathogenic**
		**Location**	**ACADS mutation** **(protein variation)**	**Location**	**ACADS mutation** **(protein variation)**			
**Short-chain acyl-CoA dehydrogenase deficiency (SCADD)**									
1	M	Exon 10	c.1130C>T (p.P377L)	Exon 9	c.1031A>G (p.E344G)	None		MIS/MIS	P
2	F	Exon 9	c.1031A>G (p.E344G)	Exon 2	c.164C>T (p.P55L)	None		MIS/MIS	P
3	F	Exon 9	c.1031A>G (p.E344G)	Exon 8	c.973C>T (p.R325W)	None		MIS/MIS	P
4	M	Exon 9	c.1031A>G (p.E344G)	Exon 10	c.1148G>A (p.R383H)	None		MIS/MIS	P/LP
5	M	Exon 9	c.1031A>G (p.E344G)	Exon 9	c.1031A>G (p.E344G)	ACADSB	c.961C>T (p.Q321^*^)	MIS/MIS/MIS	P
6	M	Exon 9	c.1031A>G (p.E344G)	Exon 2	c.164C>T (p.P55L)	None		MIS/MIS	P
7	M	Exon 9	c.1031A>G (p.E344G)	Exon 10	c.1153G>A (p.A385T)	None		MIS/MIS	P/VUS
8	F	Exon 8	c.991G>A (p.A331T)	Exon 9	c.1031A>G (p.E344G)	None		MIS/MIS	VUS/P
9	M	Exon 3	c.233G>A (p.G78E)	Exon 6	c.781T>A (p.F261I)	None		MIS/MIS	VUS
**Isobutyryl-CoA dehydrogenase deficiency (IBDD)**									
1	M	Exon 9	c.1060G>A (p.A354T)	Exon 10	c.1176G>T (p.R392S)	None		MIS/MIS	P
2	M	Intron 9	c.1092+1G>A (/)	Exon 2	c.173G>A (p.R58Q)	None		SP/MIS	LP/VUS
3	M	Exon 3	c.286G>A (p.G96S)	Exon 9	c.1000C>T (p.R334C)	None		MIS/MIS	LP
4	M	Exon 4	c.413del (p.N138Mfs^*^36)	Exon 10	c.1176G>T (p.R392S)	ACAD8	c.567+8C>T (/)	DEL/MIS/SP	LP/VUS/VUS
5	M	Exon 3	c.286G>A (p.G96S)	Exon 3	c.213G>T (p.E71D)	None		MIS/MIS	LP/VUS
6	M	Exon 5	c.553C>T (p.L185F)	Exon 9	c.1190T>C (p.L397P)	None		MIS/MIS	LP
7	M	Exon 3	c.344G>A (p.G115D)	Exon 3	c.374T>C (p.I125T)	None		MIS/MIS	VUS
8	F	Exon 7	c.725C>G (p.P242R)	Exon 9	c.988C>T (p.R330W)	None		MIS/MIS	VUS

*PS: M, male; F, female; MIS, missense mutation; LP, likely pathogenic; N, no; P, pathogenic; VUS, variant of uncertain significance. The meaning of the gray values was Suspected case*.

* *was nonsense mutation with no amino acid*.

**Figure 2:**
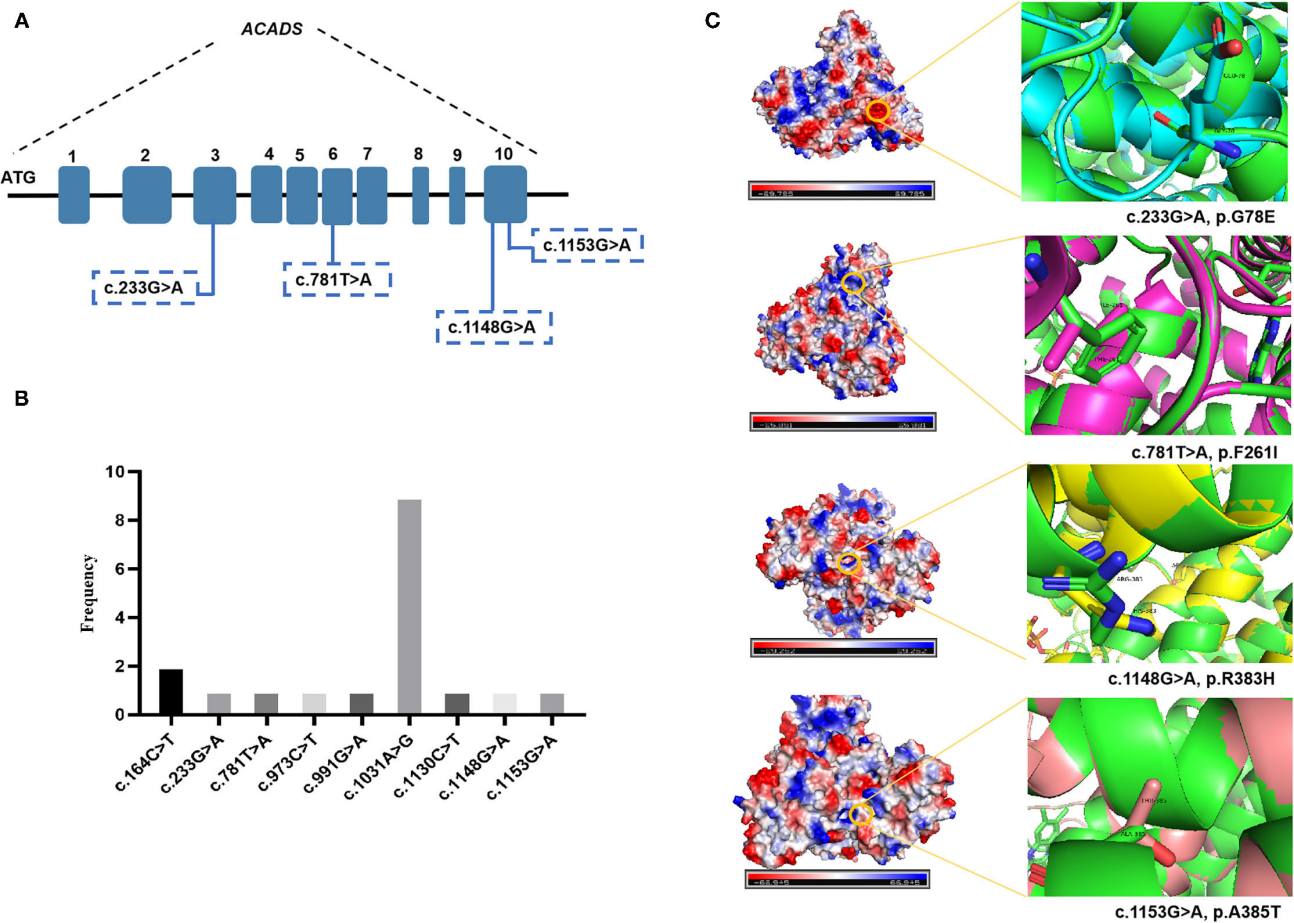
Bioinformatic analyses of *ACADS*-encoded proteins: three-dimensional structural modeling of mutant SCAD proteins. The green color represents the wild-type protein. **(A)** Four novel *ACADS* gene variants. **(B)** Frequency of different *ACADS* gene variants. **(C)** Swiss modeling and surface charge structure of novel SCAD variants.

**Figure 3:**
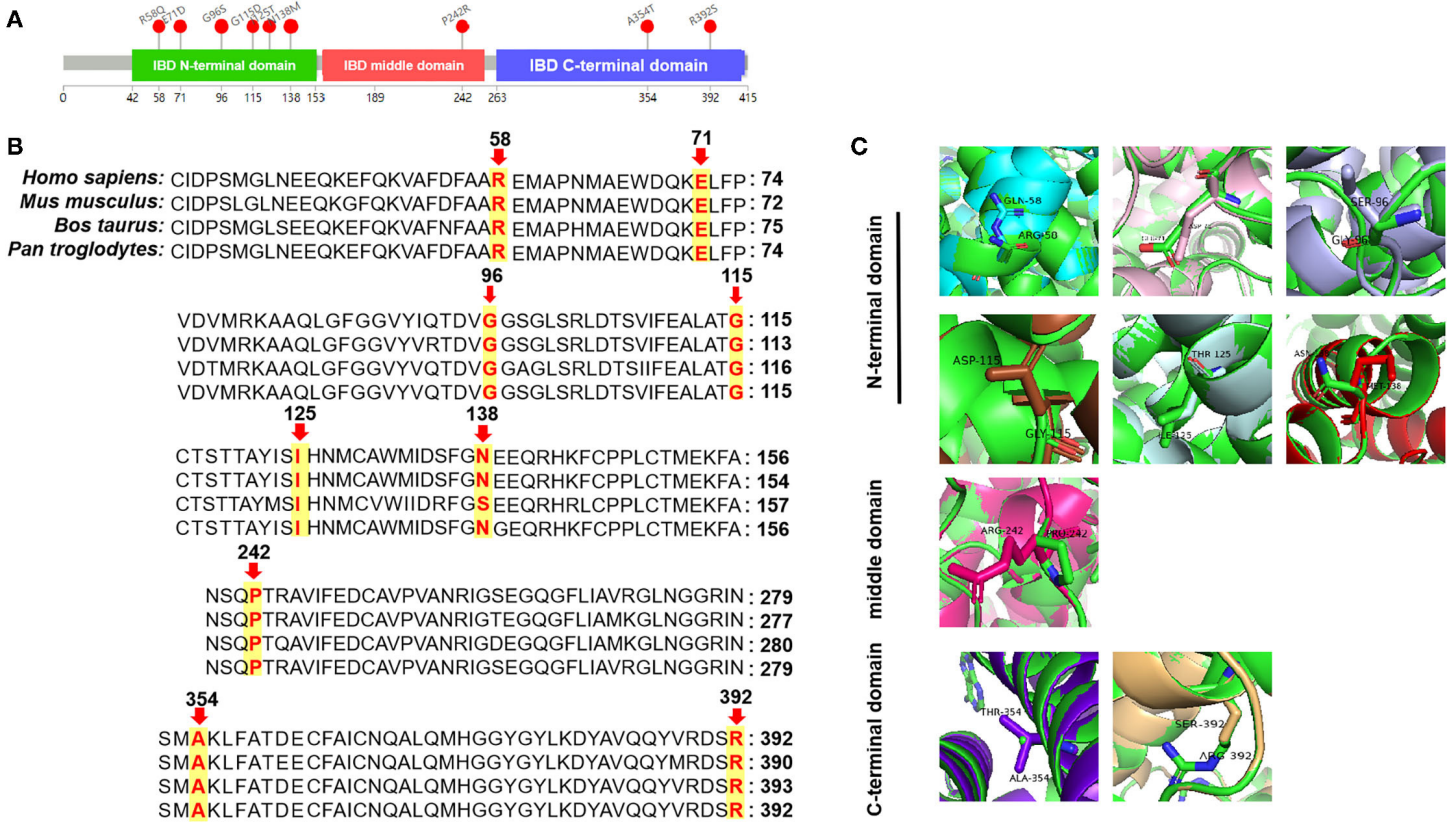
Bioinformatic analyses of *ACAD8*-encoded proteins: three-dimensional structural modeling of mutant IBD proteins. **(A)** Structure of the IBD protein with mutation sites marked. **(B)** Analysis of amino acid conservation in variant sites among several different species. **(C)** Swiss modeling of novel IBD protein variants. Arg-58 (Gln colored in cyan), Glu-71 (Asp in pink), Gly-96 (Ser in light blue), Gly-115 (Asp in brown), Ile-125 (Thr in pale cyan), and Asn-138 (Met in red) are located in the N-terminal domain; Pro-242 (Arg in magenta) is located in the middle domain; Ala-354 (Thr in purple) and Arg-392 (Ser in wheat) are located in the C-terminal domain.

**Figure 4:**
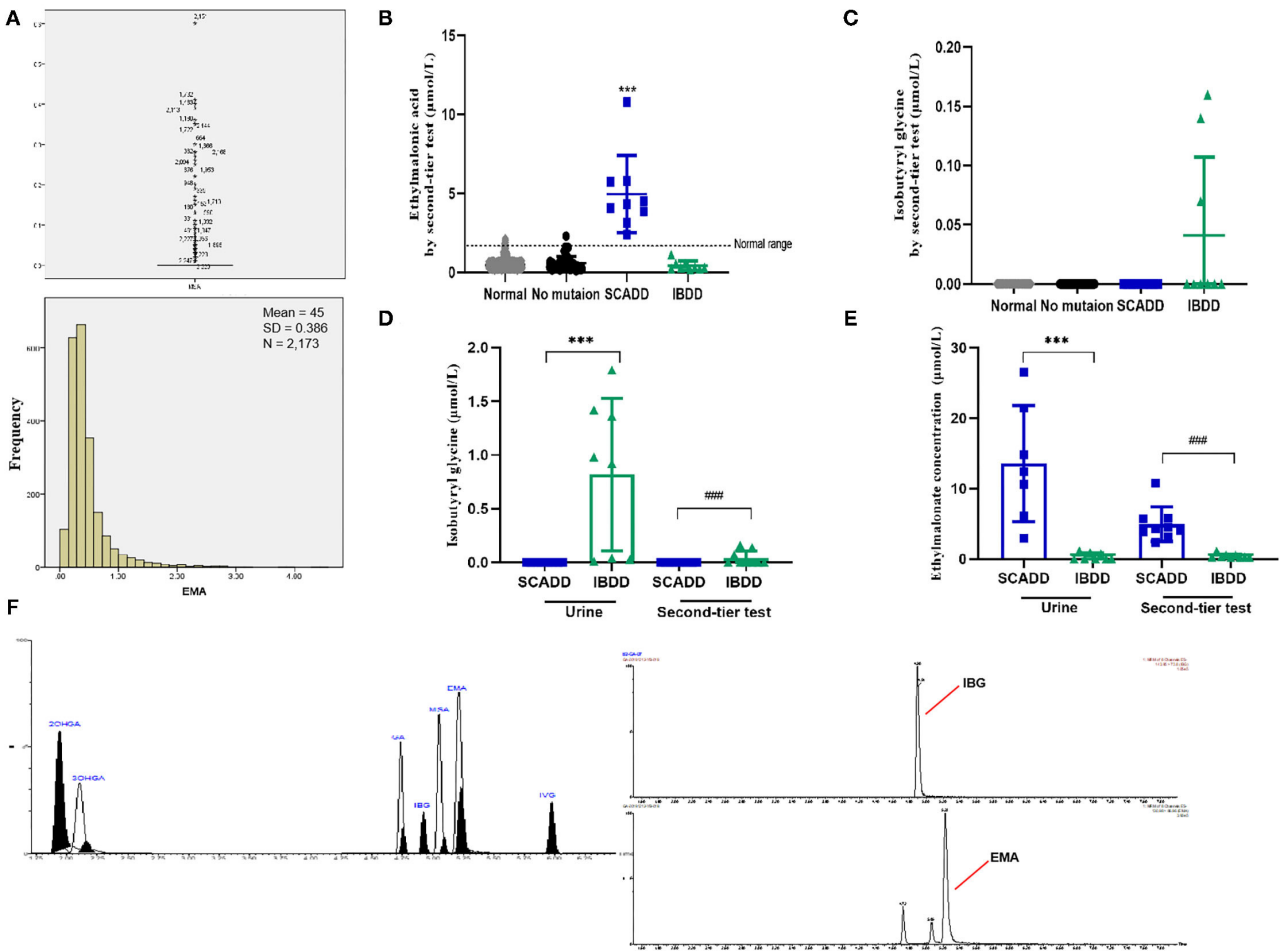
Metabolite levels identified by the second-tier test and urinary GC/MS. **(A)** Statistical analyses of outliers and normal distribution results for EMA. **(B)** Second-tier test for EMA in DBSs. **(C)** Second-tier test for IBG in DBSs. **(D)** EMA concentrations in individuals with SCADD/IBDD determined by the second-tier test and urinary GC/MS. **(E)** IBG concentrations in individuals with SCADD/IBDD determined using the second-tier test and urinary GC/MS. **(F)** Chromatographic separation of several acylcarnitines. Calibration curve, points constructed from 11 standardized acylcarnitines. The following acylcarnitine species were extracted from the calibration curve point chromatogram: 2OHGA, 2-hydroxyglutaric aciduria; 3OHGA, 3-hydroxyglutaric aciduria; GA, glutaric acidemia; IBG, isobutyryl-glycine; and IVG, isovaleric acidemia. ^***^p < 0.001, ^*###*^*p* < 0.001.

DNA sequence analysis of 76 genes for NBS revealed a larger number of variants in confirmed SCADD/IBDD cases, and two associated genes (*ACADS* and *ACAD8*) were identified. The molecular spectrum comprised nine different variants in *ACADS*: five have previously been reported, and the other four are novel. The most common variant was c.1031A>G (p. E344G), which is located in exon 9 of *ACADS*; this variant was found in 9/18 alleles (50%). Despite the limited number of cases, *in silico* and protein modeling analyses suggested that novel *ACADS* gene mutations may affect protein function (Figure 2). By combining analysis with biochemical data, all the individuals carrying novel variants were tested for increasing C4 concentration by MS/MS and urinary GC/MS and novel variants confirmed in Xuzhou, China, were considered disease-associated mutation types. In addition, 15 *ACAD8* gene variants were detected in eight infants (Table 2). All variants with the exception of c.567+8C> T and c.1092+1G> A, are located in exons. Nine previously unreported variants are absent in disease databases, and exhibit extremely low frequencies in the global population, as illustrated in Figure 3. Similarly, the alignment of *ACAD8* sequences revealed that the amino acids at positions 58, 71, 96, 115, 138, 242, 354, and 392 are highly conserved (Figure 4F). Protein modeling showed that all of the novel variants with the exception of that at position 242 are located in the α-helixes, and alterations in properties may be attributed to abnormal folding. Nonetheless, replacement of a hydrophobic proline with a basic arginine at position 242 of IBD may also result in a lack of cis peptide bonding. According to ACMG/AMP guidelines, the above variants are considered VUSs (PM2+PP3) based on sequence variation interpretation (26).

Not unexpectedly, no significant differences in C4, C4/C2, and C4/C3 values were found between SCADD/IBDD cases and the suspected cases based on biochemical data (Figures 1C–E). However, in the confirmed SCADD group, the mean C4 concentration obtained from initially measured data was 1.50 μmol/L, which was not significantly different from that of the confirmed IBDD group (C4 = 1.54 μmol/L). Correspondingly, C4/C2 and C4/C3 ratios in the confirmed SCADD group were 0.095 and 1.12, respectively, similar to the mean values of 0.12 and 1.22 found for the confirmed IBDD group (Table 1). The initial MS/MS screening used to evaluate the clinically differential detection of SCADD/IBDD was indeterminate; a second-tier test may be performed for differential screening of the latter.

### Generalizability of a Second-Tier Approach: Cutoffs for SCADD and IBDD

Notably, two presumptive outliers of EMA (4.32 and 4.53 μmol/L) are depicted in Figure 4A. However, the D/R ratio was ≤1/3, and the two values were not outliers. Previous studies have shown no significant difference in metabolite levels based on sex. As indicated in Supplementary Table 4, despite the slightly more extreme values, the 99.0th percentile (2.12 μmol/L) was far lower than the cutoff value previously reported by the Mayo Clinic Laboratory. Nevertheless, the 99.7th percentile (2.89 μmol/L) was chosen as the cutoff for second-tier screening, which resulted in percentages of 0.67% for the suspected positive rate (Table 3). Sixteen cases exceeded the EMA cutoff of 2.89 mmol/L, including all primary and secondary targets (*n* = 4). Because of the lack of further analysis, the IBG concentration in all of the negative individuals was zero.

**Table 3:** The positive rates of different percentile cut-offs with EMA by second-tier test.

**Percentile**	**Percentile value for EMA** **(μmol/L)**	**Positive cases in total samples**	**Total**	
			**Numbers**	**Rate**
P95	1.16	140	2,394	5.85
P97.5	1.51	76	2,394	3.17
P98	1.63	65	2,394	2.72
P99	2.12	38	2,394	1.59
P99.5	2.69	23	2,394	0.96
P99.7	2.89	16	2,394	0.67

### Second-Tier Testing by UPLC-MS/MS Separation of C4 Acylcarnitine in Confirmed Individuals

The second-tier test for EMA and IBG was performed using additional punched specimens from initial DBSs, and these two analytes were assessed in addition to the initial MS/MS analytes. As shown in Table 4, UPLC-MS/MS to detect EMA and IBG levels in DBSs validated methodologically based on the recovery rate and intraday/interday precision. A second-tier test was designed with three replications, and quality controls with high, median, and low levels were used to assess the experimental stability, recovery rates, and intraday/interday precision were calculated with narrower confidence intervals. Thus, the results of the UPLC-MS/MS analysis for detecting the level of IVG in DBSs were credible. The second-tier test identified 16 samples as likely SCADD and 3 as likely IBDD from March 2019 to March 2020 (Table 5). Because an uncertain category in NBS would be reported as requiring clinical follow-up by MS/MS and second-tier testing, the second-tier test would eliminate 91.98% (218/237) of the initial positive results. At this stage, molecular genetic analyses had not been confirmed.

**Table 4:** The method verification of precision for UPLC-MS/MS to detect the level of isovalerylglycine in dry filter paper.

		**Standard (μmol/)**	**Measured values (μmol/)**			**Recovery rate**			**Intra-day precision (CV, %)**			**Inter-day precision** **(CV, %)**
**Level**			**001**	**002**	**003**	**001**	**002**	**003**	**001**	**002**	**003**	**001~003**
**EMA**												
L1		1513.89	1995.64	1981.16		109.16%	99.43%	96.08%	5.57%	3.94%	3.91%	4.61%
			2106.34	1943.95	1832.06							
			2030.70	2160.15	1912.85							
			1888.17	2073.71	1846.81							
			2167.05	2112.21	1762.71							
			2193.91	2039.63	1716.02							
	AVE		2063.64	2051.80	1822.17							
	STD		114.88	80.79	71.22							
	CV		5.57%	3.94%	3.91%							
L2		3784.72	3939.50	4420.63		100.83%	103.85%	103.67%	4.56%	3.36%	4.14%	4.83%
			4045.93	4291.56	4049.64							
			4295.63	4432.48	4328.85							
			4315.25	4747.57	4347.26							
			4317.82	4491.88	4291.52							
			4448.94	4477.88	4569.80							
	AVE		4227.18	4477.00	4291.22							
	STD		192.59	150.32	177.59							
	CV		4.56%	3.36%	4.14%							
L3		7569.45	8557.90	7764.72		103.31%	98.20%	107.90%	3.57%	5.27%	2.90%	4.57%
			8514.53	8712.15	8433.62							
			8393.95	7887.24	8840.04							
			7918.17	8165.90	8394.36							
			8088.75	7864.01	8169.04							
			7913.35	7484.57	8722.02							
	AVE		8231.11	7979.77	8535.25							
	STD		294.21	420.58	247.30							
	CV		3.57%	5.27%	2.90%							
**IBG**												
L1		1722.24	2099.18	1690.68		110.80%	103.00%	109.72%	8.85%	6.92%	7.71%	8.54%
			2013.18	1708.22	1889.31							
			1945.06	1894.32	2049.78							
			1822.34	1629.58	1953.41							
			1952.44	1943.30	2007.91							
			1617.75	1776.82	1763.51							
	AVE		1908.33	1773.82	1889.67							
	STD		168.84	122.70	145.65							
	CV		8.85%	6.92%	7.71%							
L2		3444.48	3749.45	3776.37		103.33%	104.95%	108.02%	4.12%	9.18%	5.71%	6.86%
			3494.96	3461.14	4057.27							
			3399.41	3009.65	3802.29							
			3499.25	3734.91	3752.28							
			3736.01	3804.43	3703.79							
			3476.33	3904.27	3431.81							
	AVE		3559.24	3615.13	3720.67							
	STD		146.68	331.69	212.35							
	CV		4.12%	9.18%	5.71%							
L3		17222.38	18790.36	16709.48	1	104.61%	102.55%	102.44%	3.70%	3.29%	11.64%	3.50%
			18541.89	18325.95	17562.62							
			18163.84	17873.06	20756.06							
			17934.65	17854.75	16409.67							
			16893.19	17245.79	19463.55							
			17771.21	17961.98	15831.08							
	AVE		18015.86	17661.84	17642.34							
	STD		667.13	581.90	2054.23							
	CV		3.70%	3.29%	11.64%							

**Table 5:** Results of second-tier test compared to initial screening by MS/MS and urinary GC/MS (2019.03–2020.03).

**Clinical category**	**Genetic analysis**	**Testing method**		
		**Initial screening**	**Second-tier test**	**Urinary testing**
		**MS/MS**	**UPLC-MS/MS**	**GC/MS**
SCADD	*ACADS*	–	16	4
IBDD	*ACAD8*	–	3	3
Uncertain	–	237	–	–
Test report time/days		3–7	7–10	20
Total number		237	237	7

Assessment of the clinical worth of using the second-tier test results was evaluated for the *ACADS* and *ACAD8* genotypes. Ethylmalonate is used as the diagnostic metabolite for SCADD because it can be markedly elevated and specific. Figure 4D illustrates box plots of EMA levels between the second-tier test and urinary detection;focuses on IBG, Figure 4E shows the different concentrations in cases with elevated C4. Overall, using a second-tier test for EMA and IBG allowed better separation between individuals with SCADD and individuals with IBDD. Indeed, the incorporation of EMA or IBG testing as a marker for SCADD and IBDD would have eliminated 91.98% (218/237) of unnecessary referrals with 100% sensitivity. Given thatcases of elevated IBG were identified in the limited screening population, this second-tier test for IBDD may not be justified. However, IBG analysis is likely to distinguish IBDD from other C4-associated disorders because this assay is characterized by specific metabolites, and their elevation suggests a different diagnosis. An additional feature of the second-tier test was the observation that this analysis minimized turnaround time without resampling and involved short preparation and chromatography times compared with those needed for urinary GC/MS (Figure 4).

We have also followed confirmed individuals with SCAD/IBD deficiency, all diagnosed through NBS by MS/MS. Several individuals were without testing in our center and we might follow up by phone (Supplementary Table 5). One patient with SCADD (no. 2) had speech delay at 3 years of age. Majority of patients with SCADD or IBDD have had normal growth and development. After a mean period of follow-up of 2.7 years (range 8 months−4 years and 2 months), we have not observed any complications that may be related to the disorder.

## Discussion

Acylcarnitine profile analysis is performed for biochemical detection of disorders of mitochondrial FAO and organic acid metabolism (27). At the start of NBS using MS/MS, clinical screening of SCADD or IBDD was primarily based on the biochemical quantification of C4, but the overlap in C4 levels between healthy and affected neonates renders C4 a poor discriminator. Butyl carnitine is also associated with several other related metabolic disorders, as mentioned above including EE, GA2, and FIGLU, which exhibit low testing specificity and sensitivity (28, 29). The use of acylcarnitine ratios, such as C4/C2, improves sensitivity, but routine screening methods do not provide further distinguishment, and the differential rate for C4-associated disorders remains poor (24). This poor differentiation necessitates further diagnostic detection, which may result in an unbalanced cost-benefit ratio for NBS and might impose an unnecessary emotional burden on the families of unaffected neonates (24). Based on the current understanding, the initial screening results in this retrospective study suggest that most recalled cases screened by MS/MS do not need resampling after a second-tier test, which emphasizes the importance of optimizing analyses with second-tier tests to determine which individuals are truly at risk for SCADD or IBDD clinical disorders.

Of interest, diagnostic testing with molecular genetic analysis revealed the occurrence of SCADD in nine neonates and IBDD in seven, but only three neonates with IBDD were distinguished by second-tier screening before DNA sequencing. However, the stability of the second-tier analytes EMA and IBG were within narrower confidence intervals. As shown in Table 4, the average recovery of the 2 s-tier testing parameters was nearly 100%, with inter- and intraday-UPLC-MS/MS methodologies of 4.67 and 6.30%, respectively. The endogenous matrix and common metabolites did not interfere with the analysis. Although C4 concentration testing was performed in the primary screening panel by MS/MS, metabolic disorders associated with elevated C4, such as SCADD and IBDD, are difficult to distinguish by initial NBS screening. Indeed, the positive predictive values (PPVs) of the primary screening targets using C4, C4/C2, and C4/C3 as markers were 3.38% (8/237) for SCADD and 1.69% (4/237) for IBDD. Overall, second-tier screening using UPLC-MS/MS is an efficient approach for improving the specificity of NBS, and all neonates with SCADD and half of those with IBDD were directly identified by the second-tier test using DBSs. In general, reflexive analysis of the same screening samples with more specific markers eliminates the need to contact parents to bring in their newborn for another sample collection, and the second-tier testing results typically override the initial NBS results. We retrospectively measured EMA and IBG in 2,394 DBS samples that included positive samples with elevated C4 identified by initial NBS, and the second-tier positive samples exceeded the EMA and IBG cutoffs of 2.89 and 0 μmol/L, respectively (Supplementary Table 4). Our results indicate that the incorporation of second-tier testing for SCADD/IBDD differential screening would have eliminated 91.89% of unnecessary referrals. Sixteen individuals were labeled as likely to have SCADD and three as likely to have IBDD. Moreover, second-tier testing is unlikely to prolong the turnaround time because the assay is performed with initial DBS samples without the need for sample recollection. Second-tier testing that measured markers other than C4 improved the differential screening rate of SCADD and IBDD with 100% specificity.

Combining analyses of data from the initial MS/MS screening, second-tier biochemical testing, and *ACADS*/*ACAD8* genotyping from DNA sequencing allowed for fine-tuning of the second-tier test to suggest that several of the initial positive MS/MS screenings that were clinically recalled cases could be excluded. The implementation of such testing prior to referral would reduce the number of individuals who require follow-up and would eliminate 91.89% of the false-positive results that are screened for C4-assiocated disorders in NBS. This discussion of performance improvement includes cases initially considered based on the MS/MS results as showing elevations in C4 and were distinguished as SCADD or IBDD by UPLC-MS/MS. Nevertheless, the present study focused on the important question of the differential detection of C4-elevated cases. Some infants with SCADD or IBDD, two rare metabolic disorders, may have no clinical symptoms; other conditions, such as IBDD, are differentially identified via specific metabolites, such as IBG. Given the uncertainty of SCADD/IBDD, a reasonable clinical course would be to clinically follow only those infants as carrying identified with two rare variants in *ACADS*/*ACAD8*. The use of a second-tier test by the NBS laboratory would avoid unnecessary referrals of disorder carriers or unaffected cases. Another ability of the second-tier test is the focus on true-positive individuals who need immediate identification to achieve improved outcomes. Clinicians should be aware that the use of second-tier testing before referral may eliminate most cases from the referral burden of a screening center.

In the present study, eight individuals from a Chinese population were diagnosed with SCADD, and the estimated incidence of SCADD was 1:58,716 at a single NBS center. The incidence of SCADD detected by MS/MS varies greatly by region, for example, 1:70,000 in Germany, 1:45,466 in southern Italy, 1:50,000 in Denmark, and 1:292,451 in California. In our study, the treatable metabolic disease IBDD showed a rare incidence of approximately 1:78,288 in a Chinese population in Xuzhou, China. Of note, the initial C4 concentration and the C4/C2 and C4/C3 ratios in our samples did not differ significantly from the reference range in the healthy, SCADD and IBDD groups (Figure 1). Therefore, our study shows that differential screening methods are necessary for clinical measurement.

Our study was performed with a large-scale Chinese population, though DNA sequencing was only performed only for individuals suspected of having SCADD/IBDD. Urinary GC/MS should be expanded in NBS following initial positive MS/MS results. Regardless, there are challenges with urinary GC/MS testing has challenges with regard to cost and turn-around time, and it is not a panacea. Certainly, many individuals in the population of our clinic carry a single non-pathogenic variant, particularly a benign variant. If available for these cases, a second-tier test would likely increase ability to distinguish individuals with clinical SCADD/IBDD from carriers because the latter do not need follow-up and do not exhibit biochemical abnormalities.

Previous reports of SCADD have described affected patients with multiple signs, including hypoglycemia, developmental delay, lactic acidosis, hypotonia, seizures, and cardiomyopathy, who exhibit variable responses to treatment and different outcomes (30–32). Of particular note, the most frequent autosomal recessive variant of the *ACADS* gene in our center was c.1031A>G (p. E344G) in exon 9. An abnormally folded SCAD protein, as discussed in more detail above, may aggregate in cells, resulting in cellular toxicity. Abnormal accumulation of organic acids may be due to loss of SCAD enzymatic activity, which increases the risk of acute metabolic acidosis and physiological stress. The confirmed *ACADS* variants were all missense and included four unreported types. Based on different bioinformatics analyses, the c.233G>A (p.G78E) and c.1148G>A (p.R383H) variant proteins display a similar unstable structure with surface charge changes (Figure 2). The novel variants c.781T>A (p.F261I) and c.1153G>A (p.A385T) were classified genetically as being related to a steric hindrance effect and lacking the catalytic properties of the wild-type enzyme (33).

Fewer than a dozen cases of IBDD are described in the literature, and the majority of these patients are well, though some need carnitine supplementation. Molecular screening identified all seven neonates with compound heterozygous *ACAD8* variants, and homozygous c.567+8C>T or c.1092+1G>A variants in introns were detected in two infants (34, 35). Based on the present clinical data, IBDD is a rare metabolic disease in our region. These six novel mutations in a Chinese population suggest that other mutations are shared across different ethnic groups. However, a significant weakness of our study is that the cohort was drawn from the screening spectrum, which has the limitation of focusing only on neonates in Xuzhou, China. Mutational analysis may aid in the prediction of SCAD and IBD activity but cannot forecast the severity of clinical presentations, which is highly unpredictable. Therefore, asymptomatic individuals remain at risk for future episodes.

## Conclusion

Our study successfully evaluated the integration of EMA and IBG analyses into routine newborn C4 screening and distinguished SCADD/IBDD from other C4-associated disorders (36). Due to technical advances and increasing knowledge of metabolites in disorders, a second-tier test may be a reasonable approach for differential detection to reduce the recall rates of MS/MS. The use of second-tier screening significantly improved the differential rate of C4-associated metabolic disorders, with 100% sensitivity (13). Moreover, the improved specificity eliminated 96.41% of false positives and incidental findings. The results also demonstrated that sequencing of a targeted cohort generates data that can substantially improve NBS by fine-tuning the second-tier test. Fortunately, a second-tier test would also assist in the follow-up of positive cases, particularly as it may be performed within a rapid time frame and without sample recollection. This robust method determined EMA/IBG with a high differential rate and may also be applied to monitor patients under treatment (24).

## Data Availability Statement

The original contributions presented in the study are included in the article/Supplementary Materials, further inquiries can be directed to the corresponding author/s.

## Ethics Statement

The studies involving human participants were reviewed and approved by the Ethics Committee of the Affiliated Xuzhou Maternity and Child Health Care Hospital of Xuzhou Medical University reviewed and approved the protocol (Committee's reference numbers: [2019] No. 8 and [2021] No. 07). Written informed consent to participate in this study was provided by the participants' legal guardian/next of kin.

## Author Contributions

WZ and MG: conception and design of study. WZ and HL: acquisition of data. WZ, HL, and HC: analysis and/or interpretation of data. WZ: drafting the manuscript. WZ and ZJ: revising the manuscript critically for important intellectual content. All authors contributed to the article and approved the submitted version.

## Funding

This work was supported by grants from Jiangsu Province Maternal and Child Health Project (Grant number: F201912) and Xuzhou Science and Technology Program (Grant number: KC19028).

## Conflict of Interest

The authors declare that the research was conducted in the absence of any commercial or financial relationships that could be construed as a potential conflict of interest.

## Publisher's Note

All claims expressed in this article are solely those of the authors and do not necessarily represent those of their affiliated organizations, or those of the publisher, the editors and the reviewers. Any product that may be evaluated in this article, or claim that may be made by its manufacturer, is not guaranteed or endorsed by the publisher.

## Abbreviations

C4: butyl carnitine
EMA: ethylmalonate
IBG: isobutyryl-glycine
DBSs: dried blood spots
NBS: newborn screening
SCADD: short-chain acyl-CoA dehydrogenase deficiency
IBDD: isobutyryl-CoA dehydrogenase deficiency
EE: ethylmalonic encephalopathy
GA2: glutaric acidemia type II
FIGLU: formiminoglutamic aciduria
MS/MS: tandem mass spectrometry
UPLC-MS/MS: Ultra-performance liquid chromatography tandem mass spectrometry
GC/MS: gas chromatography/mass spectrometry.
