# Supplementary material for: Quantification of Differential Metabolites in Dried Blood Spots Using Second-Tier Testing for SCADD/IBDD Disorders Based on Large-Scale Newborn Screening in a Chinese Population
Source: Front Pediatr. 2021 Nov 19;9:757424. doi: 10.3389/fped.2021.757424 (PMC8639864; doi:10.3389/fped.2021.757424)
Supplement: Supplementary file 1 [file Data_Sheet_1.docx]

| Supplementary Table 1. The disease spectrum and corresponding genes of panel for molecular genetic diagnosis of metabolic disorders. | | | |
| --- | --- | --- | --- |
| Name | Gene | Name | Gene |
| Phenylketonuria | *PAH* | Glutaracidemia type I | *GCDH* |
| Tetrahydrobiopterin deficiency | *PSQDPR GCH1*  *SPR*  *PCBD1* | 3-hydroxy-3-methylglutaric acidemia | *HMGCS2* |
| Maple syrup urine disease | *BCKDHA BCKDHB DBT* | Multiple carboxylase deficiency | *HLCS*  *BTD* |
| Homocysteinemia | *DLD*  *CBS*  *MTR MTHFR* | 3-methylcrotonyl-coenzyme A carboxylase deficiency | *MCCC1 MCCC2* |
| Citrullinemia type Ⅰ | *ASS1* | β-ketothiolase deficiency | *ACAT1* |
| Citrullinemia type II | *SLC25A13* | Methylmalonic acidemia | *MUT*  *MCEE MMAA MMAB MMACHC MMADHC LMBRD1* |
| Tyrosinemia | *FAH*  *TAT*  *HPD* | Propionic acidemia | *PCCA PCCB* |
| Hyperprolinemia | *PRODH ALDH4A1* | Malonic acidemia | *MLYCD* |
| Argininemia | *ARG1* | Pyruvate carboxylase deficiency | *PC* |
| Ornithine transcarbamylase deficiency | *OTC* | Pyruvate dehydrogenase E3-Binding protein deficiency | *PDHX* |
| Carbamoyl phosphate synthetase I deficiency | *CPS1* | Pyruvate dehydrogenase phosphatase deficiency | *PDP1* |
| Argininosuccinic aciduria | *ASL* | Pyruvate decarboxylase deficiency | *DHA1* |
| Histidinemia | *HAL* | 2-methylbutyryl-Coenzyme A dehydrogenase deficiency | *ACADSB* |
| Hypermethioninemia | *MAT1A AHCY GNMT ADK* | Glucose-6-phosphate dehydrogenase deficiency | *G6PD* |
| Hyperornithinemia-hyperammonemia-homocitrullinuria syndorme | *SLC25A15* | Glycogen storage disease | *G6PC SLC37A4 SLC17A3 GAA*  *AGL*  *GBE1 PYGM* |
| Non-ketotic hyperglycinemia | *GLDC AMT GCSH* | Murcopolysaccharidosis | *IDUA*  *IDS*  *GNS HGSNAT NAGLU SGSH GALNS GLB1*  *ARSB*  *GUSB HYAL1* |
| Hyperornithinemia due to ornithine aminotransferase deficiency | *OAT* |  |  |
| Aspartylglucosaminuria | *AGA* |  |  |
| Trosine hydroxylase deficiency | TH |  |  |

| Supplementary Table 2. Parameter settings of second-tier analyte testing by UPLC-MS/MS | | | | | |
| --- | --- | --- | --- | --- | --- |
| Ion source | Type | Desolventizzazione | Cone gas flow | Temperature |  |
| ESI | Anion | 800 L/hr | 50 L/hr | 500 ℃ |  |
|  | Q1 | Q3 | Cap/KV | Cone/V | CE/V |
| EMA | 130.98 | 86.96 | 3 | 16 | 10 |
| IBG | 143.95 | 73.80 | 3 | 25 | 10 |

| Supplementary Table 3. The internal standard of EMA and IBG | | | |
| --- | --- | --- | --- |
| Name | Concentration (nmol/L) | Retention time (min) | Response |
| EMA | 3784.725 | 5.182 | 5.52E+04 |
| IBG | 3444.48 | 4.92 | 2.21E+04 |

| Supplementary Table 4. Statistical percentile of EMA concentrations by UPLC-MS/MS for negative samples (μmol/L) | | | | | | | | | | |
| --- | --- | --- | --- | --- | --- | --- | --- | --- | --- | --- |
|  | **Total** | **Mean****±SD** | **Max** | **P50** | **P95** | **P97.5** | **P98** | **P99** | **P99.5** | **P99.7** |
| **EMA** | 2308 | 0.46±0.39 | 4.53 | 0.35 | 1.16 | 1.51 | 1.63 | 2.12 | 2.69 | 2.89 |

| **Supplementary Table 5. Biochemical data and clinical features in patients with SCADD/IBDD with follow-up** | | | | | | |
| --- | --- | --- | --- | --- | --- | --- |
| No. | Age at last f/u  (y, m) | Clinical development | Growth | Metabolic parameters by MS/MS at recent visit | | |
|  |  |  |  | C4  (μmol/L) | C4/C2 | C4/C3 |
| **Short-chain acyl-CoA dehydrogenase deficiency (SCADD)** | | | | | | |
| 1 | 1y, 1m | Normal | Normal | 2.32 | 0.07 | 2.34 |
| 2 | 3y | Speech delay | Normal | 1.93 | 0.11 | 1.97 |
| 3 | 8m | Normal | Normal | 1.88 | 0.1 | 1.07 |
| 4 | 4y, 2m | Normal (f/u with phone) | Normal | - | - | - |
| 5 | 3y, 4m | Normal (f/u with phone) | Normal | - | - | - |
| 6 | 3y, 7m | Normal (f/u with phone) | Normal | - | - | - |
| 7 | 3y, 2m | Slight development delay, below average | Normal | - | - | - |
| 8 | 2y, 10m | Normal (f/u with phone) | Normal | - | - | - |
| **Isobutyryl-CoA dehydrogenase deficiency (IBDD)** | | | | | | |
| 1 | 1y, 1m | Normal | Normal | 3.4 | 0.18 | 1.28 |
| 2 | 3y, 10m | Normal | Normal | 0.39 | 0.03 | 0.31 |
| 3 | 3y, 10m | Normal | Normal | - | - | - |
| 4 | 3y, 4m | Normal | Normal | 1.45 | 0.11 | 0.95 |
| 5 | 2y, 4m | Slight development delay, below average | Normal | 2.34 | 0.15 | 1.29 |
| 6 | 1y, 3m | Normal | Normal | 2.29 | 0.16 | 1.89 |
| y, year; m, month; f/u, follow-up | | | | | | |
